# Der „Musterhitzeschutzplan für den organisierten Sport“ des Bundesministeriums für Gesundheit – Hintergrund, Aufbau und Inhalte

**DOI:** 10.1007/s00103-026-04222-w

**Published:** 2026-04-01

**Authors:** Sven Schneider, Susanna Sophie Hinn, Juliane Mirow, Andrea Nakoinz

**Affiliations:** 1 Medizinische Fakultät Mannheim, Zentrum für Präventivmedizin und Digitale Gesundheit (CPD), Universität Heidelberg, Alte Brauerei, Röntgenstraße 7, 68167 Mannheim, Deutschland; 2 Deutscher Olympischer Sportbund (DOSB), Frankfurt am Main, Deutschland; 3 Medizinische Fakultät Mannheim, Zentrum für Präventivmedizin und Digitale Gesundheit (CPD), Universität Heidelberg, Mannheim, Deutschland; 4 Universität Potsdam, Potsdam, Deutschland

**Keywords:** Hitze, Hohe Temperatur, Klimawandel, Sport, Prävention, Heat, Hot temperature, Climate change, Sports, Prevention

## Abstract

Sowohl im Wettkampfsport als auch im Breiten- und Schulsport lässt sich die Exposition gegenüber hohen Außentemperaturen häufig nicht vermeiden. Das Bundesministerium für Gesundheit veröffentlichte 2025 den „Musterhitzeschutzplan für den organisierten Sport“, dessen Ziel es ist, Sporttreibende vor hitzebedingten Gesundheitsrisiken zu schützen. Der Plan richtet sich an Sportvereine, Sportverbände und Sportstättenbetreiber und soll ihnen helfen, durch verhältnispräventive Maßnahmen nicht nur die Sporttreibenden, sondern auch Trainer*innen, Kampfrichter*innen, Funktionär*innen, Servicekräfte und Zuschauer*innen vor den gesundheitlichen Gefahren durch Hitze zu bewahren. Er soll dazu beitragen, dass nicht jeder Verein ein eigenes Hitzeschutzkonzept von Grund auf neu konzipieren muss. Dieser Artikel berichtet über den Erstellungsprozess des Plans und geht auf dessen Gliederung, Inhalte sowie die Einordnung in die bisherige Präventionslandschaft ein.

In Anlehnung an Empfehlungen der Weltgesundheitsorganisation und des Bundesministeriums für Umwelt, Klimaschutz, Naturschutz und nukleare Sicherheit umfasst der Musterhitzeschutzplan Maßnahmen: 1) zur Vorbereitung auf den Sommer, 2) zum Schutz während des Sommers, 3) bei akuten Hitzeperioden sowie 4) zur mittel- und langfristigen Anpassung. Zur Vorbereitung sollten Ablaufpläne erstellt und verantwortliche Personen benannt werden. Zu den technisch-baulichen Maßnahmen zählen die Installation von Sonnensegeln und die Verschattung von Auswechselbänken. Bei akuten Hitzewellen können Sprühnebelanlagen für Kühlung sorgen. Außerdem können Trainings- und Spielzeiten verlegt und bei Wettkämpfen zusätzliche Einwechselungen und Trinkpausen erlaubt werden. Zu den langfristigen Maßnahmen zählen die Reorganisation des Wettkampf- und Ligabetriebs und die Etablierung eindeutiger Absage- und Verlegungsregime.

**Zusatzmaterial online:**

Zusätzliche Informationen sind in der Online-Version dieses Artikels (10.1007/s00103-026-04222-w) enthalten.

## Abstract (en)

Both in competitive sports and in recreational and school sports, exposure to high outdoor temperatures often cannot be avoided. In 2025, the Federal Ministry of Health published the “Model Heat Protection Plan for Organized Sport”, which aims to protect athletes from heat-related health risks. The plan is aimed at sports clubs, sports associations, and sports facility operators, and is intended to help them use structural preventive measures not only to protect athletes, but also coaches, referees, officials, service staff, and spectators from the health hazards caused by heat. It is designed to ensure that not every club has to develop its own heat protection concept from scratch. This article reports on the development process of the plan and discusses its structure, content, and its place within the existing prevention landscape.

Based on recommendations from the World Health Organization and the Federal Ministry for the Environment, Climate Action, Nature Conservation and Nuclear Safety, the heat protection plan includes measures 1) to prepare for summer, 2) to protect during summer, 3) for acute heat waves, and 4) for medium- and long-term adaptation. Preparation should include drawing up process plans and designating responsible persons. Technical and structural measures include installing sunshades and shading substitute benches. During acute heat waves, misting systems can provide cooling. In addition, training and match times can be rescheduled, and extra substitutions and drink breaks may be allowed during competitions. Long-term measures include reorganizing competitions and league operations, and establishing clear cancellation and rescheduling regimes.

## Hintergrund

Auch in Deutschland zeigen sich die gesundheitlichen Folgen des Klimawandels: Zu den direkten Folgen zählen zunehmende Hitzewellen und andere Extremwetterereignisse sowie vermehrte UV-Strahlung. Als indirekte Folgen des Klimawandels sind Veränderungen in Ökosystemen zu verstehen, die unter anderem im Zusammenhang mit zunehmenden allergenen Belastungen und Infektionsrisiken stehen. Hinzu kommen Auswirkungen auf die mentale Gesundheit [1, 2].

Laut Weltgesundheitsorganisation (WHO) zählen Sporttreibende zu den besonders betroffenen Bevölkerungsgruppen für klimaassoziierte Gesundheitsrisiken [3]. Sowohl im Wettkampfsport als auch im Breiten- und Schulsport lässt sich die Exposition gegenüber diesen klimaassoziierten Gesundheitsrisiken oftmals nicht vermeiden. So ist u. a. bei den meisten Sportarten im Freien ein Ausweichen auf Hallen per se nicht möglich. Im Leistungs- und Breitensport lassen langfristig terminierte Spielpläne (Spielpaarungen im Ligabetrieb) und Saisonhöhepunkte (Qualifikationen, Meisterschaften, Endspiele) kaum Spielräume. Und im Schulsport sind Stundenpläne und Curricula einzuhalten. In allen Settings erschwert die limitierte Verfügbarkeit von Sporträumen (Trainings- und Belegungspläne) ein Ausweichen vor den o. g. Gesundheitsrisiken zusätzlich. Dies gilt insbesondere hinsichtlich hoher Außentemperaturen und der erwarteten Zunahme von Hitzewellen.

Innerhalb des „organisierten Sports“ betreiben mehr als 15 Mio. Menschen Sport im Freien (engl. „outdoor sports“). Dies entspricht 19 % der Gesamtbevölkerung in Deutschland und 54 % der in Deutschland aktuell 29 Mio. Menschen, die unter dem Dach des Deutschen Olympischen Sportbunds (DOSB) in rund 86.000 Sportvereinen aktiv sind [4]. Ein adäquater Hitzeschutz sollte in diesem Zusammenhang nicht nur die aktiven Sportler*innen berücksichtigen, sondern auch alle anderen am Sport beteiligten Personen, wie z. B. Trainer*innen, Kampfrichter*innen, Servicepersonal und Zuschauer*innen, die bei Training und Wettkampf teilweise stundenlang Hitzerisiken ausgesetzt sind.

Presseberichte aus den unterschiedlichen Sportarten und Regionen zeigen eindrücklich, wie wichtig wirksame Hitzeschutzkonzepte im Sport sind, um die oben genannten Beteiligten vor den Folgen anhaltender Hitzeexposition zu schützen: Bei den Bundesjugendspielen in Fulda Ende Juni 2022 mussten beispielsweise 10 Schüler*innen nach Kreislauf- und Hitzebeschwerden vom Rettungsdienst behandelt werden, 3 von ihnen im Krankenhaus, was zum vorzeitigen Abbruch der Veranstaltung führte [5]. Beim „Kiel-Lauf“ Anfang September 2024 führten Temperaturen über 28 °C zu zahlreichen medizinischen Notfällen unter den Läufer*innen. Es mussten 3 Teilnehmende reanimiert werden und Dutzende erhielten vor Ort oder im Krankenhaus medizinische Versorgung [6]. Dass auch der Hallensport betroffen ist, zeigt etwa der Abbruch des Finales der Frauen um die Deutsche Tischtennis-Meisterschaft im Sommer 2025. Bei einer Innentemperatur von rund 39 °C drohte u. a. eine minderjährige Spielerin zu kollabieren, was die Gastmannschaft zur Aufgabe zwang [7].

Hitze stellt auch ohne sportliche Betätigung eine gesundheitliche Belastung für den Körper dar. Um eine Körperkerntemperatur von 37 °C aufrechtzuerhalten, kommt es im Rahmen der Thermoregulation zum Abstrahlen von Wärme über die Erweiterung von Blutgefäßen in der Haut sowie zum Abkühlen durch Verdunstung beim Schwitzen. Bei hohen Außentemperaturen und hoher Luftfeuchtigkeit sind diese physiologischen Mechanismen eingeschränkt. Ist die Abgabe von Wärme unzureichend und erfolgt keine aktive Kühlung von außen, kann es zu einem Aufstauen der Wärme im Körper und zu einem gefährlichen Anstieg der Körperkerntemperatur kommen. Diese sogenannte Hyperthermie verursacht im Anfangsstadium häufig Kopfschmerzen und Übelkeit und kann im Extremfall zu einem Hitzschlag mit einer Körperkerntemperatur über 40 °C und konsekutivem Multiorganversagen führen. Laut einem Review verliefen gemäß der dort berücksichtigten Daten rund 49 % der erfassten Hitzschläge auch bei zügiger intensivmedizinischer Behandlung tödlich [8]. Zusätzlich belastet Hitze alle Organsysteme. Es kommt zu vermehrten Schlaganfällen und Herz-Kreislauf-Erkrankungen. Die Nierenfunktion sowie zentrale physiologische Regelkreise im Körper werden beeinträchtigt.

Diese temperaturassoziierten Risiken verstärken sich grundsätzlich mit dem Ausmaß körperlicher, z. B. sportlicher Aktivität. Die daraus resultierende Ermüdung kann koordinative und kognitive Fähigkeiten (Propriozeption, Balance, motorische Kontrolle und posturale Stabilität) beeinträchtigen, was die Wahrscheinlichkeit für Sportunfälle und -verletzungen erhöht. Auch erhöhen sich das Risiko einer Hyperthermie, oft in Verbindung mit einer Dehydratation, und das Risiko für einen sogenannten anstrengungsbedingten Hitzschlag. Laut einem internationalen systematischen Review sollen 7 % aller medizinischen Vorfälle während organisierter Sportveranstaltungen auf Hitze zurückzuführen sein [9].

Ein besonderes Augenmerk ist bei Hitze auf Sporttreibende zu legen, die Medikamente einnehmen, welche die hitzespezifische Anpassungsfähigkeit verringern. Dazu gehören beispielsweise die meisten Psychopharmaka. Diese wirken anticholinerg und reduzieren damit die Fähigkeit, zu schwitzen. Im Übrigen haben auch paralympische Athlet*innen durch ein Zusammenspiel multipler Faktoren ein besonders hohes Risiko für hitzeassoziierte Erkrankungen [10].

Präventiv wird Sportler*innen eine schonende Hitzegewöhnung durch regelmäßige Belastung über mindestens 2 Wochen empfohlen. Dann reagiert der menschliche Körper auf anhaltende thermophysiologische Belastungen mit einer Akklimatisation, welche durch bessere Hautdurchblutung, höheres Körperwasser- und Herzzeitvolumen sowie früheres und intensiveres Schwitzen gekennzeichnet ist. Angesichts der hierzulande oft kurzfristig auftretenden und meist nur wenige Tage anhaltenden Hitzewellen ist eine optimale Akklimatisation im Trainings- und Wettkampfbetrieb aber oft nicht möglich [11, 12]. Dies gilt insbesondere für den Breitensport.

Auf die durch den Klimawandel immer wahrscheinlicher werdenden Hitzeperioden hat das Bundesministerium für Gesundheit (BMG) im Sommer 2023 in einem ersten Schritt mit dem „Hitzeschutzplan für Gesundheit“ reagiert [14]. Da dem Bund aufgrund des föderalen Systems in Deutschland kein durchgreifendes Organisationsrecht beim Hitzeschutz zusteht, hat das BMG in der Folge mehrere Musterhitzeschutzpläne explizit als sogenannte Bundesempfehlungen veröffentlicht. Die Erarbeitung erfolgte jeweils gemeinsam mit Verbänden und Expert*innen aus den jeweiligen Fachgebieten. Das erste Dokument dieser Art war der „Musterhitzeschutzplan für Krankenhäuser“ [15]. Es folgten der „Musterhitzeschutzplan für Apotheken“ sowie der „Musterhitzeschutzplan für ambulante psychotherapeutische Praxen“, die zusammen mit dem Musterhitzeschutzplan für den organisierten Sport im Jahr 2025 veröffentlicht wurden [16, 17].

Dieser Beitrag berichtet über den Erstellungsprozess des Musterhitzeschutzplans für den organisierten Sport und geht auf dessen Gliederung, Inhalte und Limitationen ein. Es erfolgt eine Einordnung in die bisherige Präventionslandschaft. Weitere direkte und indirekte Folgen des Klimawandels, die im Sport ebenfalls relevant sind, werden andernorts ausführlich behandelt [13]. Dies sind unter anderem erhöhte Risiken für Hautschäden durch UV-Strahlung und für Lebensmittelinfektionen [2]. Als erwünschter Nebeneffekt reduzieren nichtsdestotrotz viele der im Musterhitzeschutzplan präsentierten Empfehlungen auch die UV-Exposition (etwa durch Verschattungsmaßnahmen) respektive Infektionsrisiken (etwa durch Hygiene und Kühlketten beim Catering).

## Ziele und Entwicklung des Musterhitzeschutzplans

### Ziel und Zielgruppen.

Übergeordnetes Ziel des Musterhitzeschutzplans für den organisierten Sport (siehe Onlinematerial) ist, alle am Breitensport beteiligten Personen vor hitzebedingten Gesundheitsrisiken zu schützen. Im Sinne eines verhältnispräventiven Ansatzes soll diese Handreichung die stets zusätzlich notwendigen individuellen, also verhaltenspräventiven Maßnahmen ergänzen, die jede*r Sportreibende situationsadäquat zum eigenen Schutz ergreifen sollte. Demzufolge richtet sich der Musterhitzeschutzplan primär nicht an den*die individuelle*n Sportler*in, sondern explizit an die verantwortlichen Institutionen, namentlich Sportvereine, Sportverbände und Sportstättenbetreiber. Dieser Zielgruppe soll der Plan helfen, durch zusätzliche verhältnispräventive Maßnahmen Sporttreibende, hauptamtlich Mitarbeitende und freiwillig Engagierte, z. B. Trainer*innen, Kampfrichter*innen, Funktionär*innen und Servicekräfte, ebenso wie Zuschauer*innen vor hitzebedingten Gesundheitsrisiken in Training und Wettkampf zu schützen. Dabei sind die im Plan enthaltenen Maßnahmen setting- und situationsadäquat zu bewerten und an die lokalen mikroklimatischen Gegebenheiten, die Herausforderungen und Bedürfnisse der jeweiligen Sportart sowie die Spezifika der betreffenden Veranstaltungsform anzupassen [18].

### Entwicklungsprozess.

Der Musterhitzeschutzplan für den organisierten Sport greift auf Vorarbeiten zurück. Er basiert auf einem 15-seitigen Musterhitzeschutzplan, der im Rahmen des Hamburger Hitzeaktionsplans 2024 erarbeitet wurde [18]. In einem umfassenden strukturierten Beteiligungsverfahren wirkten seinerzeit verschiedene Institutionen mit, unter anderem die Deutsche Allianz Klimawandel und Gesundheit (KLUG) e. V., die Behörde für Arbeit, Gesundheit, Soziales, Familie und Integration (Sozialbehörde) der Freien und der Hansestadt Hamburg, der Deutsche Olympische Sportbund (DOSB), der Hamburger Sportbund (HSB) e. V., der Hamburger Sport-Verein (HSV) e. V. sowie die Medizinische Fakultät Mannheim der Universität Heidelberg.

Darüber hinaus wurden für den Musterhitzeschutzplan des BMG wissenschaftliche und sportpraktische Veröffentlichungen und Handlungsempfehlungen berücksichtigt, die seit jener ersten Version des Musterhitzeschutzplans erschienen waren [19–24]. Ebenfalls in Form eines Beteiligungsverfahrens wurden im Frühjahr 2025 praxisnahe Rückmeldungen zur ersten Fassung des Musterhitzeschutzplans von den Mitgliedsorganisationen des DOSB eingeholt und in die Bundesempfehlung eingearbeitet. Nicht zuletzt flossen die Ergebnisse einer einschlägigen, wissenschaftlichen Delphi-Studie in den Musterhitzeschutzplan ein. In diese Studie wurden in einem transdisziplinären Verfahren zum einen 24 Ärzt*innen mit fachärztlicher Expertise in Dermatologie, innerer Medizin, Allergologie, Sportmedizin, Infektiologie und Toxikologie sowie 24 Expert*innen aus den 8 mitgliederstärksten Outdoorsportverbänden des DOSB eingebunden. Zu den Expert*innen zählten unter anderem Teilnehmer*innen an Olympischen Spielen, Weltcups und Weltmeisterschaften, Bundestrainer*innen und Spitzenfunktionär*innen ebenso wie Sportler*innen, Trainer*innen und Vereinsvertreter*innen aus dem Breitensport. Sie wurden zu praxiserprobten respektive -tauglichen Hitzeanpassungsmaßnahmen in ihren jeweiligen Sportarten befragt [25].

Die Überarbeitung und Aktualisierung des ursprünglichen Musterhitzeschutzplans aus dem Jahr 2024 wurde durch das BMG als Beitrag zur Weiterentwicklung des oben erwähnten „Hitzeschutzplans für Gesundheit“ unterstützt und von diesem am 03.06.2025 als Bundesempfehlung veröffentlicht.

### Autor*innen und Kosten.

Autor*innen des Musterhitzeschutzplans waren die Verfasser*innen des vorliegenden Beitrages (AN und JM, seinerzeit Mitarbeiter*innen bei KLUG e. V., SvS, Medizinische Fakultät Mannheim der Universität Heidelberg, sowie SH, DOSB). Die Erarbeitung des Musterhitzeschutzplans erfolgte ohne Zuhilfenahme künstlicher Intelligenz und ohne jegliche finanzielle oder anderweitige Gegenleistung des BMG.

## Aufbau und Inhalte des Musterhitzeschutzplans

Die Gliederung des Musterhitzeschutzplans folgt den Empfehlungen zur Erstellung von Hitzeaktionsplänen für Deutschland [26]. Diese Handlungsempfehlungen sehen 4 Phasen vor, die sich auch in dem vorliegenden Plan widerspiegeln: Konkret werden Maßnahmen 1) zur *Vorbereitung auf den Sommer* vorgeschlagen, die wiederum 2) *im Sommer* konkretisiert und während 3) einer *akuten Hitzeperiode* verschärft werden. Der Musterhitzeschutzplan schließt mit Vorschlägen zur 4) *mittel- und langfristigen Anpassung* für den organisierten Sport.

Zusätzlich zu dieser zeitlichen Gliederung folgt der Plan einer Gliederung in baulich-technische Maßnahmen („Sportstätte“), in organisatorische Maßnahmen („übergreifende Organisation“) sowie in Maßnahmen bei Training und Wettkampf („Trainingsbetrieb“, „Wettkämpfe und Veranstaltungen“). Als Querschnittsmaßnahmen folgen Empfehlungen zu Information, Kommunikation und Edukation der zu schützenden Zielgruppen. Auch diese zweite Gliederungsebene schließt sich an entsprechende Vorarbeiten [19, 22, 23] sowie an das „TOP“-Prinzip im klassischen Arbeitsschutz [27, 28] an: Demgemäß sind arbeitsplatzspezifische Risiken zunächst durch technisch-bauliche Maßnahmen (T) zu verhindern. Reicht dies nicht aus, so sind am Arbeitsplatz zusätzliche organisatorische (zeitliche oder räumliche) Maßnahmen (O) zu implementieren. Personenbezogene Maßnahmen (P) dienen schließlich dazu, verbleibende Restrisiken durch individuellen Schutz weiter zu reduzieren. Kommunikative Maßnahmen greifen nicht nur aktuelle Vorarbeiten auf [19, 22, 23], sondern orientieren sich darüber hinaus an den Handlungsempfehlungen der WHO und des Bundesministeriums für Umwelt, Klimaschutz, Naturschutz und nukleare Sicherheit (BMUKN; [26]), bei denen die Kommunikation von Risiken und Anpassungsmaßnahmen ebenfalls ein Kernelement darstellt.

Die zweidimensionale Gliederung des Musterhitzeschutzplans soll es Personen aus den Zielgruppen erlauben, sich in spezifischen Situationen (z. B. bei einem zu planenden Jugendfußballturnier oder anstehenden Leichtathletik-Kreismeisterschaften an einem Wochenende mit akuter „Hitzewarnstufe 1“ des Deutschen Wetterdienstes, DWD) schnell innerhalb des Planes orientieren zu können. Da mittel- und langfristige Maßnahmen dabei die geringste Priorität haben, befinden diese sich am Ende des Plans. Damit folgt der Plan der Struktur der übrigen, oben erwähnten Musterhitzeschutzpläne. Insgesamt sind 90 Einzelmaßnahmen enthalten, ausgewählte Beispiele sind in Tab. 1 dargestellt. Im Folgenden werden diese Maßnahmen in ihrer zeitlichen Abfolge kurz skizziert:

**Tab. 1:** Beispielhafte Auswahl an Einzelmaßnahmen aus dem Musterhitzeschutzplan für den organisierten Sport [44]

	Sportstätte	Übergreifende Organisation	Trainingsbetrieb	Wettkämpfe und Veranstaltungen	Information, Kommunikation und Edukation
Maßnahmen zur Vorbereitung auf den Sommer	Hitzeorientierungsplan für Innenräume und Außengelände erstellen. Darin besonders hitzeexponierte Bereiche und kühle Orte sowie Wasser-Entnahmestellen, Kühlmöglichkeiten usw. darstellen	Zuständigkeiten personell zuweisen Risiken und Maßnahmen des letzten Sommers evaluieren	Maßnahmenkatalog zum Schutz der Sporttreibenden erarbeiten	Festlegen, ob und wann Wettkämpfe bei extremer Hitze abgesagt oder verschoben werden können	Schulungsmaßnahmen vorbereiten Informationsmaterialien des DOSB, der Verbände und des BIÖG bevorraten
Maßnahmen zum Schutz während des Sommers	Temporäre Verschattung (Sonnensegel, Pavillons) installieren/aufstellen Belüftungsregime in Sporthallen anpassen	Lokale Begehung der Anlagen durchführen, um Schäden an Schutzeinrichtungen zu identifizieren	Trainingseinheiten auf verschattete Bereiche verlegen Sprühflaschen, Kühlpads, Ersatz-Sonnenschutzcreme und -Kopfbedeckung bereitstellen	Hitzeschutzkonzept im Vorfeld an Teilnehmer*innen kommunizieren Trinkwasser und Ersatz-Kopfbedeckungen, -Sonnenschutzcreme und -Trinkflaschen kostenlos ausgeben Kühlketten beim Catering beachten Zeitliche und örtliche Rotation der Kampfrichter*innen und des übrigen Personals organisieren	Schulungen zu Hitzeanpassung und Erste-Hilfe-Maßnahmen für Trainer*innen, Sportler*innen und Eltern durchführen
Spezielle Maßnahmen während akuter Hitzeperioden (inkl. Hitzewarnstufen 1 + 2)	Kühle Räume ausschildern und während des Sportbetriebes zugänglich halten	Nebelduschen und Wassersprinkler einsetzen	Trainingszeiten, -umfang und -intensität anpassen Pre‑, Per- und Postcooling-Methoden ins Training integrieren Aufmerksamkeit gegenüber Hitzesymptomen und besonders vulnerablen Sportler*innen erhöhen	Zusätzliche Hitzeschutzmaßnahmen in der technischen Besprechung abstimmen Möglichst Startzeiten, Austragungsort und Wettkampfregeln anpassen	Hitzewarnungen und Verhaltenstipps (z. B. über Stadiondurchsagen oder Anzeigetafeln) verbreiten
Maßnahmen zur mittel- und langfristigen Anpassung	Wasserspender auf der Sportanlage installieren Kühlmöglichkeiten schaffen Neben- und Verkehrsflächen entsiegeln und umgestalten	Sommerpause verlängern Winterpause verkürzen Verlagerung der Anstoß- und Startzeiten in die Morgen- oder Abendstunden	Trainingsbetrieb restrukturieren	Sportartspezifische Regelwerke und Saisonzeiten klimagerecht weiterentwickeln (Zusatzpausen, Spieldauer, Spielerwechsel, Bekleidungsvorschriften) Eindeutige Verlegungs- und Absageregime etablieren	Hitzeschutz und -adaptation in Aus-, und Weiterbildungs-Curricula der Verbände integrieren

### Maßnahmen zur Vorbereitung auf den Sommer

Entscheidend für die wirkungsvolle Reaktion auf Hitzewellen im Sommer ist die Vorbereitung. Der wichtigste Schritt ist dabei, dass Hitzeschutz als Thema erkannt und eine verantwortliche Person benannt wird. Die Zeit vor dem Sommer sollte genutzt werden, um die vorhandenen technisch-baulichen Verschattungseinrichtungen auf ihre Funktionalität zu überprüfen und gegebenenfalls zu ergänzen oder zu reparieren. Dies kann Markisen und Sonnensegel als fest installierten ebenso wie Pavillons und Sonnenschirme als mobilen Sonnenschutz betreffen. Bei der Planung sommerlicher Veranstaltungen kann unter Berücksichtigung des erwartbaren Sonnenverlaufs hier bereits darauf geachtet werden, dass Schattenbereiche auch für bewegungseingeschränkte Personen barrierefrei erreichbar sein werden und am Veranstaltungstag selektiv diejenigen Tribünen- und Zuschauerbereiche geöffnet werden, die im Schatten liegen werden. In Vorbereitung auf den Sommer kann ebenfalls ein Maßnahmenkatalog etwa in Form eines Workflows erarbeitet werden, der für den Bedarfsfall verantwortliche Personen identifiziert und das Vorgehen bei Extremwetterereignissen fixiert. Mit Blick auf den Wettkampf- und Veranstaltungskalender sollte das Thema Absagerichtlinien bedacht und besprochen werden. Mögliche Orientierungshilfen können durch die jeweiligen Fachverbände oder Landessportbünde eingeholt werden. Bei der Veranstaltungsplanung lässt sich zu diesem Zeitpunkt bereits berücksichtigen, dass Start- und Zielbereiche sowie Aufenthaltszonen in schattigen Bereichen liegen (z. B. bei Laufveranstaltungen oder Turnieren) und dass bei geplanter Bewirtung Kühlketten gewährleistet sind und das Bewirtungsangebot entsprechend an Hitze angepasst ist. Im Allgemeinen sollten die Sensibilisierung für das Gesundheitsrisiko sowie eine Integration der Inhalte in bereits bestehende Formate (z. B. Schulungsmaterialien) angestrebt werden.

### Maßnahmen zum Schutz während des Sommers

Zu Beginn des Sommers sollte das Belüftungsregime in den Sporthallen und Sportanlagen angepasst werden und etwaiger mobiler Sonnenschutz (wie Sonnensegel, Großschirme) witterungssicher vorbereitet bzw. installiert werden. Während des Trainings und bei Wettkämpfen sollte die Möglichkeit bestehen, fehlenden Eigenschutz vor Ort niederschwellig und kostenlos zur Verfügung gestellt zu bekommen: Vergessene Trinkflaschen, Sonnenschutzmittel und Kopfbedeckungen sollten ausgeliehen respektive ausgegeben und Trinkwasser zugänglich gemacht werden. Derartige Angebote erscheinen insbesondere für Kinder und Jugendliche wichtig, da diese entwicklungsbedingt regelmäßig ein geringeres Risikobewusstsein aufweisen. Darüber hinaus sind Schutzmaßnahmen in besonderem Maße für Menschen mit Behinderung relevant, da spezifische funktionelle Einschränkungen, etwa infolge eines Querschnittssyndroms oder bei Extremitätenamputationen, ebenso wie kognitive Beeinträchtigungen häufig mit einem erhöhten gesundheitlichen Risiko unter klimatisch veränderten Bedingungen einhergehen [10].

Bei Veranstaltungen im Sommer sollten die vorgesehenen Maßnahmen zum Hitzeschutz zur besseren individuellen Vorbereitung bereits vorab (etwa über die Veranstalterwebsite, Newsletter oder Push-Nachrichten) an vereinseigene und -fremde Teilnehmende (Vereine, Trainer*innen, Schiedsrichter*innen, Sportler*innen) kommuniziert werden. Vor Ort können während des Wettkampfbetriebs Lautsprecherdurchsagen oder Anzeigetafeln genutzt werden, um Temperatur, UV-Index und Verhaltenshinweise an die Teilnehmerschaft zu kommunizieren. Durch rotierende Einsatzpläne lässt sich nicht zuletzt die Exposition gegenüber Hitze und UV-Strahlung für Kampfrichter*innen, Ehrenamtliche und das Servicepersonal auf einfache Weise reduzieren.

### Maßnahmen während akuter Hitzeperioden

Während akuter Hitzeperioden, etwa bei Hitzewarnstufe 1 oder 2 des DWD, sollten die oben genannten Maßnahmen weiter ergänzt werden. Der DWD ruft Hitzewarnstufe 1, also eine „starke Wärmebelastung“ aus, wenn die gefühlte Temperatur am frühen Nachmittag einen Schwellenwert von etwa 32 °C überschreitet. Diese kann unter Berücksichtiung eines etwaigen Akklimatisationseffektes bei Ereignissen im Frühsommer etwas niedriger und im Hochsommer etwas höher liegen. Als weiteres Kriterium einer Warnung wird die nächtliche Temperatur von Innenräumen herangezogen. Überschreitet die gefühlte Temperatur am frühen Nachmittag einen Wert von 38 °C, so wird vor einer „extremen Wärmebelastung“ gewarnt und Hitzewarnstufe 2 ausgerufen. Je nach Wetterlage werden ältere Menschen sowie Stadtbewohner*innen im Rahmen der Warnungen besonders angesprochen [29].

Im Sport können Sprühnebelanlagen („Nebelduschen“) und Rasensprenger zur Nutzung von Verdunstungskälte vor Ort zum Einsatz kommen. Zudem sollten Trainer*innen, sofern es die Belegungspläne zulassen, Trainingszeiten in die frühen Morgenstunden oder auf den späten Abend verlegen und ihre Aufmerksamkeit insbesondere gegenüber besonders vulnerablen Personen erhöhen. Ebenso kann eine Anpassung von Trainingsplänen die Belastung der Sportler*innen bei extremer Hitze reduzieren. Ausdauereinheiten können durch Koordinations‑, Technik- oder Taktikeinheiten ersetzt werden. Während des Trainings ist es hilfreich, wenn Trainer*innen in ihrem Training regelmäßige Pausen zur Flüssigkeitsaufnahme, zur Kühlung etwa durch feuchte Handtücher oder Sprühflaschen und zum Erneuern des Sonnenschutzes integrieren. An heißen Tagen sollten Trainer*innen auf Symptome hitzebedingter Erkrankungen hinweisen und darauf achten. Dazu enthält der Musterhitzeschutzplan als Anlage eine Auflistung zu spezifischen medizinischen Risikogruppen [30].

Bei Wettkämpfen und Veranstaltungen unter akuter Hitze sollten im Rahmen der vor Spielbeginn i. d. R. obligatorischen „technischen Besprechung“ entsprechende Anpassungsmaßnahmen zwischen Schiedsrichter*innen bzw. Kampfrichter*innen, Trainer*innen und Sportler*innen abgestimmt werden. Je nach Setting (Freizeitturnier, Ligaspiel, Meisterschaftswettkampf) können hier zusätzliche Kühl- und Schattenpausen, zusätzliche Spielerwechsel und verkürzte Spielzeiten ebenso vereinbart werden wie eine Lockerung und/oder Anpassung der sportartspezifischen Kleidungsregeln oder eine örtliche und zeitliche Verlegung des Spiels oder des Wettkampfs.

### Maßnahmen zur mittel- und langfristigen Anpassung

Einen im Vergleich dazu deutlich längeren Vorlauf benötigen i. d. R. bauliche und technische Maßnahmen zum nachhaltigen Hitzeschutz in und auf Sportstätten und -anlagen. Diese können eine energetische Sanierung, eine Dach- oder Fassadenbegrünung, eine Eingrünung oder eine Beseitigung von Versiegelungen und Hitzeinseln umfassen. Ebenfalls eher mittelfristig lässt sich eine grundlegende Restrukturierung des Trainings- und Wettkampfbetriebs umsetzen. Hierunter fallen Überlegungen zu einer Verlängerung der Sommerpause und einer Verkürzung der Winterpause innerhalb des Ligabetriebs sowie die Verlegung von Startzeiten von sommerlichen Turnieren und Ligaspielen in die Morgen- oder Abendstunden. Abstimmungsprozesse sind ebenfalls nötig, wenn sportartspezifische Regelwerke klimagerecht angepasst werden sollen. Sportartspezifisch sind die Einführung von zusätzlichen Trinkpausen (Tab. 2) sowie die Festlegung von klaren Regularien zur Absage eines Wettkampfes denkbar. Bei der Beschaffung von Sportkleidung (Trikotsätze usw.) kann der Verein auf helle und UV-zertifizierte Stoffe achten. Flankierend wäre eine sportartspezifische und bedarfsorientierte Implementierung entsprechender Inhalte in bestehende Weiterbildungsangebote der Sportverbände wünschenswert.

**Tab. 2:** Beispiele für regulatorische Maßnahmen zum Hitzeschutz im Sport bei spezifischen Sportarten

Sportart	Beispiele für regulatorische Maßnahmen
Fußball	Nach den offiziellen Regeln des Deutschen Fußballbundes können Schiedsrichter*innen an Hitzetagen während des Spiels Trinkpausen gewähren. Je nach einschlägiger Wettbewerbsbestimmung ist hierfür eine Spielunterbrechung von maximal einer Minute vorgesehen, in der die Spieler*innen Flüssigkeit aufnehmen dürfen. Darüber hinaus gibt es die Möglichkeit, sogenannte Kühlpausen zu ermöglichen. Kühlpausen sind Spielunterbrechungen von 90 s bis maximal 3 min, die je nach Wettbewerbsbestimmungen „im Sinne des Wohlbefindens oder der Sicherheit der Spieler*innen“ bei bestimmter Witterung (z. B. hoher Luftfeuchtigkeit und hoher Temperatur) der Senkung der Körpertemperatur dienen sollen. In der Praxis werden typischerweise zusätzliche (oft 2‑minütige) Pausen nach Bedarf bei ruhendem Spiel etwa in der Mitte jeder Halbzeit durch die Schiedsrichter*innen gewährt [23, 37]
Tennis	Gemäß Wettspielordnung des Hessischen Tennis-Verbands können Spiele bei extremer Hitze unter bestimmten Bedingungen verlegt werden. Dazu muss für den Spieltag am Heimspielort die vorhergesagte Tageshöchsttemperatur mindestens 36 °C betragen. Eine entsprechende Vorhersage ist am Tag vor dem Spiel zwischen 10 Uhr und 12 Uhr einem definierten Wetterportal zu entnehmen und nachweisbar zu dokumentieren (z. B. mittels Screenshots). Die Verlegung des Wettkampfs kann dann von jeder Mannschaft auch ohne Einverständnis der gegnerischen Mannschaft in Anspruch genommen werden und muss bis spätestens 13 Uhr am Vortag dem Gegner mitgeteilt werden [45]
Golf	Der Bayerische Golfverband hat Handlungsanweisungen entwickelt, um Spieler*innen vor übermäßiger Hitze bei Turnieren zu schützen. Gemäß Turnierstatut kann ein Spieltag wegen extremer Hitze verlegt werden, wenn für den Spieltag am Heimspielort die vorhergesagte Temperatur in dem Zeitraum der letzten Startzeit zuzüglich 5 h eine bestimmte Temperatur überschreitet. Diese Temperaturen sind altersspezifisch festgelegt. Sie betragen für Spieler*innen bis zum Alter von 18 Jahren und für Spieler*innen im Alter über 65 Jahren 32 °C und für alle anderen Altersklassen 35 °C. Bei einer prognostizierten Tageshöchsttemperatur über 38 °C sieht die Empfehlung vor, ganze Spieltage zu verlegen oder die Startzeiten entsprechend anzupassen. Das Prozedere zur Dokumentation und zur Absage respektive Verlegung des Spieltags entspricht im Wesentlichen der oben dargestellten Vorgehensweise im Tennis. Der Golfverband sieht darüber hinaus ein entsprechendes Disqualifikationsregime bei missbräuchlicher Inanspruchnahme der Hitzeregelung vor [38]

## Einordnung des Musterhitzeschutzplans in die bisherige Präventionslandschaft und Limitationen

Als Dachorganisation des deutschen Sports hat der DOSB keine Weisungsbefugnis gegenüber seinen Mitgliedsorganisationen, den Spitzenverbänden der olympischen und nichtolympischen Sportarten. Die Spitzenverbände selbst wiederum sind auf Bundeslandebene eigenständig organisiert und unterschiedlich ausdifferenziert [31]. Dieses komplexe, u. a. föderal begründete Organisationsgebilde des organisierten Sports in Deutschland erschwert einen koordinierten Hitzeschutz. Bis dato fehlt ein umfassendes Konzept zur Hitzeanpassung – und zwar sowohl sportartübergreifend auf Bundesebene als auch sportartspezifisch innerhalb der jeweiligen Spitzenverbände.

Einige Verbände informieren auf ihren Webseiten über Maßnahmen zum Hitzeschutz. Diese sportartspezifischen Web- oder Print-Veröffentlichungen stellen größtenteils Informationen oder Empfehlungen für Sportler*innen und weitere Personen, also verhaltenspräventive Maßnahmen dar (z. B. ausreichende Flüssigkeitszufuhr). So informieren einige – aber längst nicht alle [32] – Verbände in Form von digitalen Newsbeiträgen über Hitzebelastung im Sport und geben Sportler*innen sportartspezifische Hinweise und Tipps (wie etwa der DAV [33], der DKV [34], die DLRG [35] und der American Football und Cheerleading Verband Nordrhein-Westfalen [36]). Detaillierte Empfehlungen haben beispielsweise der Deutsche Fußball-Bund (DFB; [37]), der Deutsche Golf Verband (DGV; [38]), der Deutsche Kanu-Verband (DKV) und der Deutsche Volleyball-Verband (DVV; [39]) herausgegeben. Bei diesen Angeboten stehen die Sensibilisierung sowie das Wissensmanagement im Vordergrund. Darüber hinaus existieren innerhalb der Sportarten vereinzelt auch erste regulatorische Konzepte zu verhältnispräventiven Maßnahmen (Tab. 2).

Was bis dato fehlte, war eine Zusammenstellung sportartenspezifisch adaptierbarer Maßnahmen zur Hitzeanpassung. Ein erster Ansatz in diese Richtung war das Projekt KLIMASPORT, welches Praxismaterialien in Form einer modularen Toolbox, inklusive Erklärvideos, von Quiz- und Rätselvorlagen, Social-Media-Kits und Plakaten für Sportvereine entwickelte [40]. Über eine Landingpage mit dem Titel „Klimawandel, Gesundheit und Sport“ ermöglicht der DOSB einen ersten Einstieg in das Thema. Dort finden sich stichpunktartig Empfehlungen an Sportler*innen, Trainer*innen, Vereine und Verbände, kurze Erklärvideos und weiterführende Literatur [41]. Dem Musterhitzeschutzplan konzeptionell am nächsten kommt eine fast zeitgleiche Veröffentlichung zu dem im Kontext Sport zweiten bedeutenden Risiko, nämlich der UV-Exposition. So hat das Bundesamt für Strahlenschutz (BfS) gemeinsam mit dem DOSB die Broschüre „Praxistipps zu UV-Schutz. Maßnahmen für Sportvereine“ veröffentlicht. Wie im Musterhitzeschutzplan werden auch dort Empfehlungen zusammengefasst und hinsichtlich der Gestaltung der Sportstätten, der Organisation des Sportbetriebes und der Information und Kommunikation gegliedert [21].

### Limitationen.

Der Musterhitzeschutzplan schlägt klassische verhältnispräventive Maßnahmen vor. Dies spiegelt sich auch in den adressierten Zielgruppen – den Sportvereinen, Sportverbänden und Sportstättenbetreibern – wider. Angesichts der rund 86.000 Sportvereine in Deutschland soll er die dort mehrheitlich ehrenamtlich Tätigen bei der klimagerechten Anpassung der Rahmenbedingungen und ihren Bemühungen zum Schutz der in ihrer Obhut befindlichen Sportler*innen unterstützen. Der Musterhitzeschutzplan soll dazu beitragen, dass nicht jeder Verein ein eigenes Hitzeschutzkonzept von Grund auf neu konzipieren muss. So sollen wertvolle Personal- und Zeitressourcen geschont werden, die dringend für die sportliche Betreuung benötigt werden. Der Musterhitzeschutzplan für den organisierten Sport betont dementsprechend bereits in der Einleitung, dass er individuelle verhaltenspräventive Maßnahmen der Sportler*innen selbst nicht adressiert. Hierzu wird in der Bundesempfehlung auf anderweitige Publikationen u. a. des DOSB verwiesen. Ebenso erstreckt er sich explizit nicht auf angrenzende Interventionsfelder, wie etwa den Klimaschutz (also die Verringerung von Treibhausgasemissionen) im Sport oder Maßnahmen zum Schutz der Sportinfrastruktur (vor Überschwemmungen, Hitzeschäden usw.).

Eine weitere Limitation stellt der Umstand dar, dass die Evidenzlage zu diesem Präventionsfeld noch lückenhaft ist und der vorliegende Maßnahmenkatalog als lebendes Dokument in einem nächsten Schritt auf Effektivität und Effizienz evaluiert werden sollte. So sind beispielsweise Maßnahmen wie das möglichst häufige Aufsuchen von Schatten und das Ermöglichen des Tragens einer Kopfbedeckung und schulterbedeckender Kleidung sowie das Auftragen von Sonnenschutzmitteln durch entsprechende medizinische Leitlinien gedeckt [42]. Andere Empfehlungen, wie etwa die Einführung zusätzlicher Trinkpausen oder die Etablierung eindeutiger Abbruchkriterien, sollten wissenschaftlich hinsichtlich konkreter Outcomes (z. B. durch die Anzahl medizinischer Notfälle) evaluiert werden. Besonders groß sind die Forschungslücken u. a. im Präventionsfeld „paralympischer Sport“ [10], in einigen Natursportarten [43] und in klassischen Hallensportarten. Auf jene Mängel in der Evidenzlage und die Notwendigkeit einer regelmäßigen Evaluation, Anpassung und Weiterentwicklung wird im Rahmen des Musterhitzeschutzplans ebenfalls hingewiesen. Deswegen wurde auch innerhalb des Musterhitzeschutzplans keine Priorisierung der Maßnahmen vorgenommen und kein Anspruch auf Vollständigkeit erhoben.

Nicht zuletzt sind die vorgeschlagenen Maßnahmen an Sportart, Disziplin und Settings zu adaptieren. An einem Sommerwochenende mit Temperaturen von über 35 °C werden die Maßnahmen bei einem Jugend-Fußballturnier unter freiem Himmel anders sein als bei einem zeitgleich stattfindenden Seniorenturnier im Tennis respektive einem Volkslauf mit 10.000 Teilnehmer*innen in der Nachbarstadt.

## Fazit

Das Thema „Hitzeschutz“ ist mittlerweile auch im Sport angekommen. Während sich Verantwortliche in anderen Settings, wie etwa am Arbeitsplatz, in Kinderbetreuungseinrichtungen und in Kommunen, dem Hitzeschutz und der Klimafolgenanpassung seit Jahren widmen, gewinnt dieses Thema im Sport im Vergleich erst seit Kurzem an Dynamik. Die Mehrzahl der zentralen Veröffentlichungen hierzu stammt aus der jüngsten Zeit.

Der im Musterhitzeschutzplan gewählte Top-down-Ansatz und der explizite Empfehlungscharakter sollen synergistisches Potenzial nutzen und den begrenzten Personalressourcen im mehrheitlich ehrenamtlich betriebenen Breitensport entgegenkommen. Andere Institutionen wie der DOSB, das Bundesinstitut für Öffentliche Gesundheit (BIÖG) und das BfS verfolgen denselben Ansatz [20, 21, 41]. Die nächsten Schritte auf dem Weg zu einem klimaangepassten, sicheren Sport sind eine sportartspezifische Implementierung, eine regelmäßige Weiterentwicklung und Evaluation der Umsetzung und eine Vernetzung mit anderen Präventionsfeldern (wie dem Arbeitsschutz, dem Schulsport usw.). So kann der Sport auch in Zeiten des Klimawandels sein umfassendes präventives Potenzial für die Gesundheit entfalten.

## Supplementary Information

Onlinematerial: Musterhitzeschutzplan für den organisierten Sport
