# Supplementary material for: The Federal Ministry of Health’s “Model Heat Protection Plan for Organized Sport”—background, structure, and content
Source: Bundesgesundheitsblatt Gesundheitsforschung Gesundheitsschutz. 2026 Apr 1;69(7):831–9. [Article in German] doi: 10.1007/s00103-026-04222-w (PMC13323785; doi:10.1007/s00103-026-04222-w)
Supplement: Supplementary file 1 — Onlinematerial: Musterhitzeschutzplan für den organisierten Sport [file 103_2026_4222_MOESM1_ESM.pdf]

BUNDESEMPFEHLUNG

Musterhitzeschutzplan für den organisierten Sport

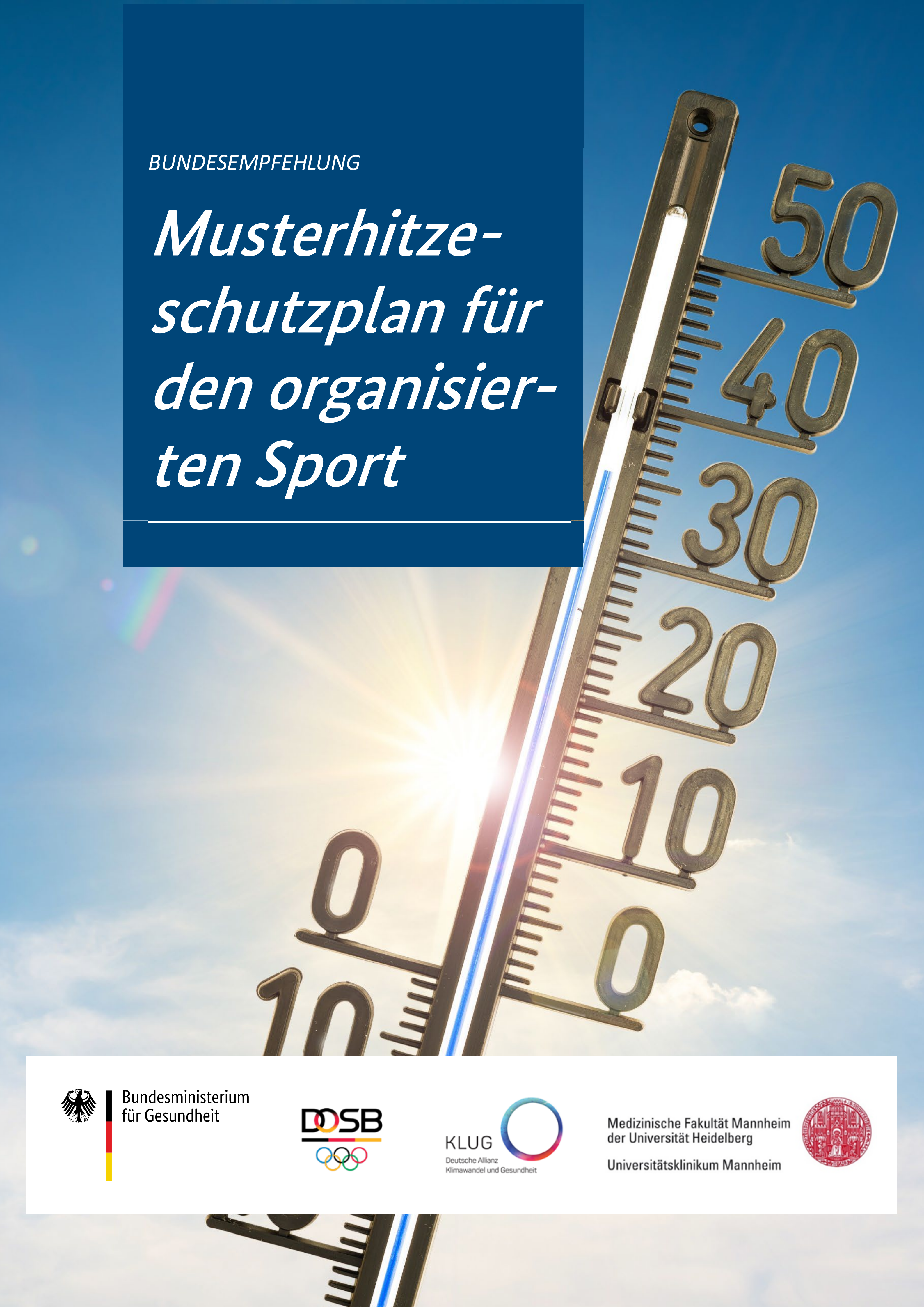

Bundesministerium für Gesundheit

DOSB

KLUG Deutsche Allianz Klimawandel und Gesundheit

Medizinische Fakultät Mannheim der Universität Heidelberg

Universitätsklinikum Mannheim

Einleitung

Laut Weltgesundheitsorganisation (WHO) zählen Sporttreibende zu den besonders betroffenen Risikogruppen für klimabedingte Gesundheitsrisiken [8]. Egal ob Sport im Freien – auf dem Sportplatz, im Park oder auf dem Wasser – oder Sport in der Halle – jede Sportart, jeder Verband und jeder Verein wird künftig mit den Auswirkungen des Klimawandels konfrontiert sein. Für den Sport zählen häufigere, längere und intensivere Hitzewellen zu den bedeutendsten Auswirkungen des Klimawandels [5, 6].

Vor diesem Hintergrund soll dieser Musterhitzeschutzplan für den organisierten Sport dabei helfen, alle Zielgruppen im Breitensport vor hitzebedingten Gesundheitsrisiken besser zu schützen. Die Adressatinnen und Adressaten dieses Musterhitzeschutzplans sind Vereine und Verbände. Ihnen soll dieser Plan helfen, Sportreibende, hauptamtlich Mitarbeitende und freiwillig Engagierte, z. B. Trainerinnen und Trainer, Kampfrichterinnen und Kampfrichter, Funktionäre und Servicekräfte, ebenso wie Zuschauerinnen und Zuschauer vor hitzebedingten Gesundheitsrisiken zu schützen. Um eine möglichst breite Wirksamkeit zu erzielen, ist es wichtig, dass bei der Umsetzung aller Maßnahmen auf Inklusion und Barrierefreiheit geachtet wird.

Um zeitnahe, niederschwellige und praktikable Empfehlungen zur Verfügung stellen zu können, fasst dieser Musterhitzeschutzplan in strukturierter Form zahlreiche Empfehlungen von Expertinnen und Experten zusammen [3, 5-8]. Dabei folgt seine Gliederung den Empfehlungen zur Erstellung von Hitzeaktionsplänen für Deutschland [2]. Es sei an dieser Stelle betont, dass die Evidenzlage zu diesem Präventionsfeld noch lückenhaft ist und der vorliegende Maßnahmenkatalog in einem nächsten Schritt auf Effektivität und Effizienz zu evaluieren ist.

Dieser Musterhitzeschutzplan für den organisierten Sport stellt eine gesundheitsspezifische Maßnahme zur Klimaanpassung dar. Deswegen werden angrenzende Interventionsfelder, wie etwa Maßnahmen zum Schutz der Sportinfrastruktur, im Folgenden nur angeschnitten.

Der vorliegende Musterhitzeschutzplan basiert auf einer Empfehlung, welche die Deutsche Allianz Klimawandel und Gesundheit (KLUG) e.V. zusammen mit der Medizinischen Fakultät Mannheim der Universität Heidelberg und dem Deutschen Olympischen Sportbund (DOSB) sowie weiteren Akteurinnen und Akteuren im Jahr 2024 veröffentlichte [4]. In der nunmehr vorliegenden Fassung wurden seitdem erschienene Veröffentlichungen (etwa des DOSB [3]) und Forschungsarbeiten [7] berücksichtigt. Zudem ermutigte der DOSB zahlreiche Sportverbände zu einer praxisbezogenen Rückmeldung zum bisherigen Musterhitzeschutzplan, welche im Folgenden ebenfalls eingearbeitet wurden.

Die Aktualisierung des Musterhitzeschutzplans wird durch das Bundesministerium für Gesundheit (BMG) als Beitrag zur Weiterentwicklung des „Hitzeschutzplans für Gesundheit" [1] ausdrücklich unterstützt und wird daher als Bundesempfehlung veröffentlicht. Die nachstehenden Maßnahmen stellen aus Sicht des BMG sinnvolle Empfehlungen dar und umfassen einen praktischen Katalog von kurzfristig realisierbaren Strategien in wesentlichen Handlungsfeldern. Sie bieten eine Orientierung für Vereine, Verbände und Sportstättenbetreibende und sind in Abhängigkeit von der Sportart, Disziplin und des Settings zu adaptieren. Die Maßnahmen sind jedoch nicht abschließend und sollten regelmäßig überprüft und bei Bedarf angepasst werden. Als verhältnispräventive Maßnahmen ergänzen sie die zusätzlich notwendigen Maßnahmen, die jeder Sportreibende zum eigenen Schutz ergreifen sollte. Individuelle Verhaltenstipps für Sporttreibende sowie weiterführende Anpassungsmöglichkeiten für Vereine und Verbände finden Sie auf der Seite des DOSB (www.dosb.de).

1. Maßnahmen zur Vorbereitung auf den Sommer

**Sportstätte**	Hitzeorientierungsplan für Innenräume und Außengelände erstellen. Darin besonders hitzebetroffene Bereiche sowie kühle Orte und Erholungsbereiche sowie Wasser-Auffüllstationen, Eisschrank, Sanitäranlagen, Erste-Hilfe-Station darstellen
	Technische Hitzeschutzmöglichkeiten an Land und auf dem Wasser auf Funktionsfähigkeit überprüfen und ggf. anschaffen, z. B. Pavillons, Sonnensegel
	Notwendige Zeit für Reparaturen und Bestellungen berücksichtigen:<br>• Jalousien<br>• Ventilatoren<br>• Sonnensegel, Sonnenschutzdächer, Schattennetze<br>• mobiler Sonnenschutz (z. B. Pavillons, Sonnenschirme mit Ständern für Coachingzonen und Kampfrichterstühle)<br>• Vernebelungsanlagen<br>• Trinkwasserspender<br>• Kühlboxen
	Verschattungsmöglichkeiten auf Nebenflächen, die für Bewegung genutzt werden, überprüfen bzw. schaffen
	Verschattung der Zuschauerbereiche bereits bei der Planung von Wettbewerben berücksichtigen
	Für verschattete Bereiche Barrierefreiheit sicherstellen
**Übergreifende Organisation**	Eine für Hitzeschutz zuständige Person benennen und hauptamtlich Mitarbeitende sowie freiwillig Engagierte über Erstellung eines Hitzeschutzkonzeptes informieren
	Zusätzlich sportart- und sportstättenspezifischen Maßnahmenkatalog für akute Hitzewarnstufen[1] erstellen
	Risiken und Maßnahmen des letzten Sommers bewerten (Evaluation)
	Kriterien für die Maßnahmenbewertung (Kenngrößen für die Evaluation) und für die laufende Aktualisierung des Hitzeschutzplans (Ereignisse, Termine) festlegen
	Vorgehen bei Extremwetterereignissen (wie beispielsweise Gewitter, Hitzewellen) bei Training, Wettkämpfen und Veranstaltungen festlegen

[1] Der Deutsche Wetterdienst (DWD) gibt amtliche Hitzewarnungen heraus: Stufe 1 (starke Wärmebelastung) und Stufe 2 (extreme Wärmebelastung). Es wird den Verantwortlichen empfohlen, DWD-Hitzewarnungen z. B. per Newsletter oder App zu abonnieren, um direkt über Hitzewarnungen informiert zu werden.

	Maßnahmen auch zum Schutz der hauptamtlich Mitarbeitenden und freiwillig Engagierten erarbeiten[2]
	Hitze in die Gefährdungsbeurteilung des Arbeitsschutzes aufnehmen
	Überbrückungskonzepte für Mangellagen (Energie, Wasser) erstellen
**Trainingsbetrieb**	Maßnahmen zum Schutz der Sporttreibenden erarbeiten
**Wettkämpfe und Veranstaltungen**	Auf hitze- und akklimatisationsgerechte Anreise- und Veranstaltungsplanung achten
	Festlegen, ob und wann Wettkämpfe bei extremer Hitze abgesagt oder verschoben werden können
	Start- und Zielbereich sowie Aufenthaltszonen in schattige Bereiche legen
	Bei erhöhtem Risiko für eine überhitzte Sportstätte, Kommune kontaktieren, um alternative Bewegungsräume zu erschließen
	Durchsagen und Anzeigen mit Informationen und Gesundheitshinweisen für kommende Wettkämpfe und Veranstaltungen erarbeiten
	Bewirtungsangebot bei Veranstaltungen im Sommer rechtzeitig abstimmen, offenes Feuer / Grill vermeiden
	Hygienestandards – auch bei ehrenamtlicher Bewirtung – zur Vermeidung von Lebensmittelinfektionen prüfen
**Information, Kommunikation und Edukation**	Sensibilisierung für Hitzerisiken und Schutzmaßnahmen sowie Erste-Hilfe-Kurse in bestehende Austauschformate und Treffen integrieren
	Kommunikationsstruktur einrichten und basierend auf den Hitzewarnstufen (interne sowie externe Kommunikation) festlegen
	Vernetzung mit Landessportbünden, Fachverbänden und anderen Vereinen, um Synergien zu nutzen und Erfahrungen auszutauschen
	Schulungsbedarf ermitteln und vorhandene Schulungskonzepte / Schulungsmaterialien nutzen[3]
	Informationsmaterialien zu Risikofaktoren, Prävention und Erste-Hilfe-Maßnahmen bei hitzebedingten Erkrankungen bestellen oder produzieren für:<br>• hauptamtlich Mitarbeitende und freiwillig Engagierte<br>• Sporttreibende und Angehörige<br>• Zuschauerinnen und Zuschauer

[2] Siehe Punkt 2 und 3 zu konkreten Maßnahmen zur Reduktion von Hitzebelastung (z. B. angepasste Arbeits-/ Trainingszeiten und -orte, technische Hitzeschutzmöglichkeiten, Verhaltenstipps etc.).

[3] Das Projekt KLIMASPORT bietet eine Toolbox an, um Fort- und Weiterbildungen zu Klimaanpassung im Sport zu unterstützen.

2. Maßnahmen zum Schutz während des Sommers

zusätzlich zu Punkt 1

**Sportstätte**	Temporäre Verschattungs- und Kühlungseinrichtungen witterungs- und unfallsicher installieren
	Belüftung der Hallen und Anlagen auf Sommer umstellen (z. B. morgendliches Querlüften)
**Übergreifende Organisation**	Im Frühsommer Begehung der Anlagen durchführen, um Schäden und Verbesserungsmöglichkeiten zu identifizieren
**Trainingsbetrieb**	Vorräte an Trinkwasser, Kühlpads, Sonnenschutzcreme, Kopfbedeckung usw. an gut erreichbaren Stellen bereitstellen und Sporttreibende an den Eigenschutz (u. a. Mitbringen von Getränken und Sonnenschutzcreme) erinnern
	Lüftungskonzepte umsetzen
	Ausreichend Pausen einrichten
	Vorbildfunktion von Trainerinnen und Trainern sowie Übungsleiterinnen und Übungsleitern nutzen
	Schattenbereiche im Training auf eigenem oder angrenzendem Gelände nutzen
**Wettkämpfe und Veranstaltungen**	Ablaufpläne für Veranstaltungen je nach Hitzewarnstufe erstellen und umsetzen; sportartspezifische Regularien beispielsweise zu Startzeiten und Grenzwerten beachten
	Vorgesehene Maßnahmen zum Hitzeschutz vorab an vereinseigene und fremde Teilnehmende kommunizieren
	Essens- und Cateringangebot an hohe Temperaturen anpassen:<br>• Auf die hygienische Lagerung von Lebensmitteln und Einhaltung von Kühlketten achten<br>• Auf Ausschank von alkoholischen, stark zuckerhaltigen, koffein- oder taurinhaltigen Getränken verzichten<br>• Mineralwasser, ungesüßten Tee und dünne Saftschorlen anbieten
	Vor Ort bereitstellen:<br>• kostenlose Trinkwasserausgabe bzw. -spender<br>• mobile Verschattung<br>• Ausgabestellen für kostenlose Sonnenschutzcreme, Kopfbedeckung, Leihsonnenbrillen (vor allem an Kinder und besonders vulnerable Personen)
	Regelmäßige, leicht verständliche Durchsagen von Temperatur, UV-Index und Verhaltenshinweisen Digitale

	Displays oder analoge Anzeigen mit den vorbereiteten Inhalten bespielen
	Kühle Orte ausweisen, Hitzeorientierungsplan auslegen oder gut sichtbar aufstellen
	(Mobile) Verschattung der Publikumsbereiche (z. B. durch tageszeitliche Steuerung geöffneter Tribünenbereiche), Auswechselbänke, Coachingzonen und Kampfrichterbereiche gewährleisten
	Hauptamtlich Mitarbeitenden, freiwillig Engagierten, Kampfrichterinnen und Kampfrichtern sowie Schiedsrichterinnen und Schiedsrichtern ausreichend Schattenpausen (lt. Arbeitsschutz: „Entwärmungsphasen“) in kühlen Räumen ermöglichen und ausreichend Flüssigkeit zur Verfügung stellen
	Beim Erstellen der Einsatzpläne auf häufigere zeitliche und örtliche Rotation von Schiedsrichterinnen und Schiedsrichtern und Kampfrichterinnen und Kampfrichtern sowie der sonstigen hauptamtlichen Mitarbeitenden und freiwillig Engagierten achten
	Erste-Hilfe-Stationen und ggf. Sanitäterinnen und Sanitäter gut sichtbar an zentraler Stelle positionieren, um sicherzustellen, dass bei hitzebedingten Erkrankungen frühzeitig reagiert werden kann
**Informationen, Kommunikation und Edukation**	Schulungen (z. B. Erste-Hilfe-Maßnahmen, Trainingsanpassung bei Hitze usw.) für Verantwortliche, hauptamtlich Mitarbeitende, freiwillig Engagierte, Trainerinnen und Trainer, Übungsleiterinnen und Übungsleiter, Sporttreibende und Eltern durchführen
	Informationsmaterialien bereitstellen z. B. vom DOSB
	Hitzerelevante Informationen streuen, wie z. B.:<br>• optimale Trainingszeiten<br>• Verwendung von Kopfbedeckung, umweltfreundlichen Sonnenschutzprodukten, nassen Handtüchern, Kühlpads, ggf. Kühlwesten<br>• rechtzeitiges Pre-, Per- und Post-Cooling<br>• Aufnahme von Wasser statt alkoholischer, koffein- oder taurinhaltiger Getränke<br>• Aufnahme leichter, wasserreicher, salzhaltiger, pflanzenbasierter und proteinarmer Nahrung in häufigeren kleineren Portionen<br>• Standorte von Trinkwasserspendern und kühlen bzw. be- oder verschatteten Orten<br>• Erkennen und Behandeln von hitzebedingten Erkrankungen

3. Spezielle Maßnahmen während akuter Hitzeperioden (inkl. Hitzewarnstufe 1 + 2)

zusätzlich zu Punkt 2

**Sportstätte**	Kühle Räume in Sportstätten und / oder in der Umgebung beschildern und während des Sportbetriebes zugänglich machen
	In Sporthallen Maßnahmen zur Senkung der Raumtemperatur und zur Belüftung einleiten
	Wärme-produzierende Geräte während der Nichtnutzung abschalten
	Exponierte Trainingsgelände beobachten, bei Überhitzung sperren und alternative Flächen ausweisen
**Übergreifende Organisation**	Ausgearbeiteten Maßnahmenkatalog je nach Hitzewarnstufe umsetzen
	Arbeitsschutzregeln prüfen und anwenden
	Erkrankte (z. B. mit Magen-Darm-Erkrankungen, Infektionskrankheiten usw.) vom Trainings- und Wettkampfbetrieb durch Trainerinnen und Trainer und Übungsleiterinnen und Übungsleiter ausschließen
	Wassersprinkler, Nebelduschen und Vernebelungsanlagen anschließen und einsatzbereit halten
**Trainingsbetrieb**	Aufmerksamkeit gegenüber besonders vulnerablen Personen[4] erhöhen und Trainerinnen und Trainer sowie Übungsleiterinnen und Übungsleiter für gesundheitliche Risiken sensibilisieren
	Trainingszeiten auf die frühen Morgen- oder späten Abendstunden verlegen und im Freien auf Mücken- und Zeckenschutz achten
	Trainingsort oder einzelne Trainingsinhalte in kühlere, verschattete Bereiche verlegen
	Häufigere und längere Schattenpausen einplanen für:<br>• Flüssigkeitsaufnahme<br>• Kühlung durch z. B. feuchte Handtücher, Sprühflaschen, Eiswürfel und / oder Kühlpads<br>• Nachcremen mit wasserfestem Sonnenschutz<br>• gemeinsame Pulskontrollen<br>• Abfragen typischer Warnzeichen und Symptome durch Trainerinnen und Trainer bzw. Übungsleiterinnen und Übungsleiter
	Ausreichende Flüssigkeitsaufnahme sicherstellen:<br>• Kostenlose Getränke bereitstellen<br>• Trinkwasser zugänglich machen

[4] Risikopersonen, wie sie im Sachstandsbericht des Robert Koch-Institutes („Klimawandel und Gesundheit" 2023, siehe Tabelle 1 im Anhang) definiert sind.

	• Trinkwasserverordnung beachten
	Trainingskleidung überprüfen und hinweisen auf:<br>• helle, schulterbedeckende Kleidung<br>• ggf. Kleidung nass / feucht anziehen<br>• zertifizierte Schutzkleidung sportartspezifisch sinnvoll einsetzen<br>• Schutzkleidung in den Pausen ablegen (z. B Helme, Protektoren)
	Trainingspläne anpassen:<br>• Trainingsumfang und Trainingsintensität reduzieren<br>• Ausdauereinheiten durch Koordinations-, Technik- oder Taktikeinheiten ersetzen
	Sporttreibende zum rechtzeitigen Auftragen von Sonnenschutzmitteln und zu Pre-, Per- und Post-Cooling-Möglichkeiten informieren und animieren
	Bei Hinweisen auf hitzebedingte Erkrankungen Training beenden, Erste-Hilfe-Maßnahmen einleiten und bei Bedarf den Rettungsdienst rufen
**Wettkämpfe und Veranstaltungen**	Sofern kurzfristig möglich, ad hoc mit den Verantwortlichen anpassen:<br>• Wettkampfzeiten auf die Morgen- oder Abendstunden verlegen<br>• Austragungsort verlegen<br>• Wettkampfregeln anpassen<br>• Kriterien zu Abbruch und Unterbrechung von Spielen und Wettkämpfen
	Hitzeschutzmaßnahmen vorab in der Technischen Besprechung mit den Kampfrichterinnen und Kampfrichtern oder Schiedsrichterinnen und Schiedsrichtern abstimmen
	Maßnahmen zur Trainingsgestaltung auf den Wettkampftag adaptieren
	Bei Erreichen der vorab definierten Abbruchkriterien Heim- und Gastmannschaften bzw. Sporttreibende schnellstmöglich informieren und auf den angekündigten Ausweichtermin verweisen
	Beim Catering koffein- und alkoholfreie Getränke und wasserreiche Kost anbieten
	Vorab definierte Ablaufpläne umsetzen
**Information, Kommunikation und Edukation**	Akute Hitzewarnungen und Verhaltenstipps an Sporttreibende verbreiten

4. Maßnahmen zur mittel- und langfristigen Anpassung

**Sportstätte**	Bauliche und technische Maßnahmen zum Hitzeschutz in Sportstätten unter Nutzung der Fördermöglichkeiten umsetzen, z. B. bezüglich:<br>• natürlicher und künstlicher Verschattung (z. B. Überdachung von Auswechselbänken)<br>• Lüftungsanlagen / Ventilationstechnik<br>• energetischer Sanierung<br>• hitzereduzierender Anstriche und Bodenbeläge<br>• hitzeresistenter, hypoallergener Dach-, Fassaden- und Geländebegrünung
	Neben- und Verkehrsflächen umgestalten:<br>• Hitzeinseln beseitigen bzw. abmildern<br>• Aufhebung von Versiegelungen und Schotterflächen
	Kühlmöglichkeiten für die hygienische Lagerung von Getränken, Proviant und Eis schaffen
	Trinkbrunnen oder Wasserspender installieren
	Reduktion von Treibhausgasen im Betrieb der Sportstätten mitdenken („Klimaschutz ist Gesundheitsschutz“)
**Übergreifende Organisation**	Grundlegende zeitliche Restrukturierung des Trainings- und Wettkampfbetriebes z. B.:<br>• Verlängerung der Sommerpause<br>• Verkürzung der Winterpause<br>• Verlagerung der Wettkämpfe, Turniere und Saisonhöhepunkte in kühlere Monate<br>• Verlagerung der Startzeiten in die Morgen- oder Abendstunden
	Sportmedizinische Untersuchungen für besonders gefährdete Sporttreibende ermöglichen
	Sportartspezifische Hitzepräventionsstrategie („Heat Policy“) verabschieden (z. B. auch für Sporttiere wie Pferde)
**Wettkämpfe und Veranstaltungen**	Sportartspezifisches Regelwerk und Saisonzeiten klimagerecht weiterentwickeln
	Wettkämpfe und Veranstaltungen außerhalb von Hitzeperioden planen
	Bei Ankündigung künftiger Wettkämpfe, Turniere und Veranstaltungen klare Absagekriterien, eindeutiges Absageregime durch

	Standard Operation Procedures (SOPs) und zeitnahen Ausweichtermin kommunizieren
**Information, Kommunikation und Edukation**	Diesen Hitzeschutzplan an die jeweiligen Anforderungen und Spezifikation jeder einzelnen Sportart und Disziplin durch die Verbände adaptieren
	Eigene Aktivitäten mit dem Hitzeaktionsplan der Kommune abgleichen und Synergien nutzen
	Curriculare Inhalte zum Hitzeschutz und zur Prävention hitzebedingter Erkrankungen zur Aus-, Fort- und Weiterbildung implementieren
	Barrierefreie Informationsmaterialen anbieten, z. B.:<br>• in verschiedenen Sprachen<br>• in Papierform sowie digital<br>• in einfacher Sprache<br>• leicht lesbar<br>• in Brailleschrift bzw. Audioformat

Quellenverzeichnis

1. Bundesministerium für Gesundheit (Hrsg.) (2023) Hitzeschutzplan für Gesundheit des BMG. www.bundesgesundheitsministerium.de/themen/praevention/hitze
2. Bundesministerium für Umwelt, Naturschutz, Bau und Reaktorsicherheit (Hrsg.) (2017) Handlungsempfehlungen für die Erstellung von Hitzeaktionsplänen zum Schutz der menschlichen Gesundheit. www.bmuv.de/fileadmin/Daten_BMU/Download_PDF/Klimaschutz/hap_handlungsempfehlungen_bf.pdf
3. Deutscher Olympischer Sportbund (2024) Klimabilanzierung und Klimaanpassung im Sport. Reihe: Sport und Umwelt, Band 39, Frankfurt. www.cdn.dosb.de/user_upload/Sportstaetten-Umwelt/DOSB-24014_BRO_Dokumentation_Bodenheimer_Symposium_low__7_.pdf
4. KLUG – Deutsche Allianz Klimawandel und Gesundheit e.V. (Hrsg.) (2024) Musterhitzeschutzplan für den organisierten Sport. www.hitze.info/wp-content/uploads/2024/12/241213_Musterhitzeschutzplan_Organisierter-Sport.pdf
5. Robert Koch Institut (Hrsg.) (2023) Sachstandsbericht Klimawandel und Gesundheit. www.rki.de/DE/Themen/Gesundheit-und-Gesellschaft/Klimawandel/Klimawandel-Gesundheit-Sachstandsbericht
6. Schneider S (Hrsg.) (2024) Gesundheitsrisiko Klimawandel – Neue Herausforderungen für Sport, Beruf und Alltag. Hogrefe Verlag, Bern. ISBN 978-3-456-86286-6. https://www.doi.org/10.1024/86286-000
7. Schneider S, Niederberger M, Kurowski L, Bade L (2024) How can outdoor sports protect themselves against climate change-related health risks? – A prevention model based on an expert Delphi study. Journal of Science and Medicine in Sport 27: 37-44. https://doi.org/10.1016/j.jsams.2023.11.002
8. Weltgesundheitsorganisation. Regionales Büro für Europa (Hrsg.) (2011) Öffentlichkeitsarbeit zur Vorbeugung von Gesundheitseffekten durch Hitze: Neue und aktualisierte Informationen für verschiedene Zielgruppen. Kopenhagen.

Anhang

Tabelle 1

Personengruppen, die bei Hitze ein größeres gesundheitliches Risiko tragen

Kategorie	Risikogruppe
**Physiologische Anpassungskapazität**	• Ältere Menschen (> 65 Jahre)<br>• Säuglinge und Kleinkinder<br>• Schwangere
**Vorerkrankungen**	• Kardiovaskuläre Erkrankungen (z. B. arterielle Hypertonie, koronare Herzkrankheit, Herzinsuffizienz)<br>• Zerebrovaskuläre Erkrankungen (z. B. Schlaganfall)<br>• Respiratorische Erkrankungen (z. B. chronisch obstruktive Lungenerkrankung (COPD), Asthma bronchiale)<br>• Stoffwechselerkrankungen (z. B. Diabetes mellitus)<br>• Neurologische Erkrankungen (z. B. Morbus Parkinson durch krankheitsbedingt beeinträchtigte Thermoregulation)<br>• Psychische Erkrankungen (z. B. Depression, Schizophrenie, Drogenabhängigkeit)<br>• Nierenerkrankungen (z. B. Niereninsuffizienz)<br>• Übergewicht<br>• Einnahme von bestimmten Medikamenten zur Behandlung der genannten Erkrankungen
**Menschen mit Behinderung**	• Körperliche Behinderungen (z. B. Rückenmarksverletzungen)<br>• Geistige Behinderungen, da sich Personen mit schweren kognitiven Einschränkungen schlechter selbst vor Hitze schützen können
**Funktionelle Einschränkungen**	• Bettlägerigkeit<br>• Unterbringung in Pflegeeinrichtung
**Sozioökonomische Faktoren**	• Soziale Isolation, insbesondere im hohen Alter<br>• Obdachlosigkeit

	• Ungünstige Wohnsituation
**Körperliche Anstrengung bei hohen Außentemperaturen**	• Im Freien Arbeitende (z. B. in der Landwirtschaft, im Bausektor)<br>• Im Freien Sporttreibende<br>• Gesundheitspersonal, v. a. in Kombination mit persönlicher Schutzausrüstung
**Beschäftigte, die ihren Arbeitsplatz auch bei hohen Innenraumtemperaturen während Hitzewellen nicht verlassen können**	• Mitarbeitende in Medizin- und Pflegeeinrichtungen, v. a. in Kombination mit persönlicher Schutzausrüstung

Quelle: Winklmayr C, Matthies-Wiesler F, Muthers S, Buchien S, Kuch B, an der Heiden M, et al. (2023) Hitze in Deutschland: Gesundheitliche Risiken und Maßnahmen zur Prävention. Journal of Health Monitoring 8(S4): 3–34. https://doi.org/10.25646/11645

Impressum

Herausgeber
Bundesministerium für Gesundheit
Referat 622 „Umweltbezogener Gesundheitsschutz, Klima und Gesundheit"
11055 Berlin
622@bmg.bund.de
www.bundesgesundheitsministerium.de

Autorinnen und Autoren
Dr. med. Andrea Nakoinz (KLUG – Deutsche Allianz Klimawandel und Gesundheit e. V.)
Juliane Mirow (KLUG – Deutsche Allianz Klimawandel und Gesundheit e. V.)
Prof. Dr. Sven Schneider (Medizinische Fakultät Mannheim der Universität Heidelberg, Zentrum für Präventivmedizin und Digitale Gesundheit (CPD))
Ressort Breiten- und Gesundheitssport & Ressort Sportstätten und Umwelt, Deutscher Olympischer Sportbund (DOSB)

Wir bedanken uns bei allen beteiligten Akteurinnen und Akteuren für die Unterstützung bei der Erstellung des Musterhitzeschutzplans.

Stand
Juni 2025

Bildnachweis
Günter Albers / Adobe Stock / Titel

Die Publikationen der Bundesregierung zum Herunterladen und zum Bestellen finden Sie unter:
www.publikationen-bundesregierung.de

URL-Verweise
Das Bundesministerium für Gesundheit verweist in seinen Publikationen über Links auf Webadressen mit Inhalten, die von Dritten angeboten werden. Solche per Querverweis erreichbaren fremden Inhalte wurden bei der Erarbeitung der Erstauflage dieser Publikation überprüft. Es ist jedoch nicht auszuschließen, dass die Inhalte im Nachhinein von den jeweiligen Anbietern verändert werden. Das Bundesministerium für Gesundheit überprüft die Inhalte, auf die in seinen Publikationen verwiesen wird, nicht ständig auf Veränderungen oder Aktualität. Sollten Sie der Ansicht sein, dass die verlinkten externen Seiten gegen geltendes Recht verstoßen oder sonst unangemessene Inhalte aufweisen, so teilen Sie uns dies bitte mit.

Diese Publikation wird im Rahmen der Öffentlichkeitsarbeit des Bundesministeriums für Gesundheit kostenlos herausgegeben und ist nicht zum Verkauf bestimmt. Sie darf weder von Parteien noch von Wahlwerbern oder Wahlhelfern während des Wahlkampfes zum Zwecke der Wahlwerbung verwendet werden. Dies gilt für Bundestags-, Landtags- und Kommunalwahlen sowie für Wahlen zum Europäischen Parlament.
